# Relevance of mortalin to cancer cell stemness and cancer therapy

**DOI:** 10.1038/srep42016

**Published:** 2017-02-06

**Authors:** Chae-Ok Yun, Priyanshu Bhargava, Youjin Na, Jung-Sun Lee, Jihoon Ryu, Sunil C. Kaul, Renu Wadhwa

**Affiliations:** 1 Department of Bioengineering, College of Engineering, Hanyang University, 222 Wangsinmi-ro, Seongdong-gu, Seoul, Republic of Korea; 2 DBT-AIST International Laboratory for Advanced Biomedicine (DAILAB), Biomedical Research Institute, National Institute of Advanced Industrial Science & Technology (AIST), Central 5-41, 1-1-1 Higashi, Tsukuba - 305 8565, Japan; 3 Graduate School of Life & Environmental Sciences, University of Tsukuba, Ibaraki- 305-8575, Japan

## Abstract

Mortalin/mtHsp70 is a member of Hsp70 family of proteins. Enriched in a large variety of cancers, it has been shown to contribute to the process of carcinogenesis by multiple ways including inactivation of tumor suppressor p53 protein, deregulation of apoptosis and activation of EMT signaling. In this study, we report that upregulation of mortalin contributes to cancer cell stemness. Several cancer cell stemness markers, such as ABCG2, OCT-4, CD133, ALDH1, CD9, MRP1 and connexin were upregulated in mortalin-overexpressing cells that showed higher ability to form spheroids. These cells also showed higher migration, and were less responsive to a variety of cancer chemotherapeutic drugs. Of note, knockdown of mortalin by specific shRNA sensitized these cells to all the drugs used in this study. We report that low doses of anti-mortalin molecules, MKT-077 and CAPE, also caused similar sensitization of cancer cells to chemotherapeutic drugs and hence are potential candidates for effective cancer chemotherapy.

Cancer is a highly complex and heterogenous disease. It is often comprised of diverse cell populations that possess different proliferative capacity, cell surface antigens, tumor forming ability and respond differently to chemotherapeutic drugs. A minority of cancer cell population, called cancer stem cells (CSC), with CD44(+/high)CD24(−/low) signature, has been identified in a large variety of cancers. These cells have been ascribed as the key determinants of malignant transformation, metastasis and multidrug resistance characteristics that form a prime cause of failure in cancer chemotherapy leading to fatality^1^,^2^,^3^. CSC are also distinguished by enriched expression of several other markers referred to as stemness factors. These include aldehyde dehydrogenase, ATP-binding cassette transporter protein-ABCG2/BCRP1, 5-transmembrane glycoprotein-CD133, and transcriptional factor OCT-4^4^,^5^,^6^,^7^,^8^,^9^. Tumor progression, especially in case of solid tumors, is often accompanied by generation of hypoxia microenvironment that in turns promotes proliferation, EMT, invasion and metastasis^10^,^11^. It has been shown that cancer cells survive during hypoxia by up-regulation of stemness factors^11^. Furthermore, CSC-enriched tumors have been shown to display chemoresistance and poor prognosis, indicating that these cells are an important target for therapeutic success^12^,^13^. In view of these reports, research on CSC biology is deemed important for understanding the process of tumorigenesis, its progression, treatment, prognosis and recurrence.

Cancer cells depend heavily on mitochondria, a key organelle for regulation of metabolism, survival and death signalings^14^. Mortalin/mtHsp70, a member of Hsp70 family, has been shown to promote proliferation, metastasis and angiogenesis, and downregulate apoptotic signaling. It has been shown to interact with p53, telomerase and hnRNP-K in cancer cells^15^,^16^,^17^,^18^,^19^,^20^,^21^. Whereas p53 is inactivated by mortalin in cancer cells, telomerase and hnRNP-K are activated and were shown to contribute to malignant transformation^22^. Mortalin was shown to inhibit p53-BAX interactions and activate AKT that are required for apoptotic signaling^18^,^23^,^24^. It was also shown to interact with complement C9, a major component of membrane attack complexes that are released in membrane vesicles from complement attacked cells accounting for resistance of cancer cells to complement-dependent cytotoxicity^25^. Increased mortalin expression was shown to mediate resistance of ovarian cancer cells to cisplatin^26^. Based on these data and our recent findings on the role of mortalin in EMT, we hypothesized that it may also be involved in cancer cell stemness. We therefore investigated several cell stemness markers and drug resistance in mortalin-overexpressing breast cancer cells. We demonstrate that mortalin-overexpressing cells were enriched with stemness markers and exhibit resistance to cytotoxicity induced by several chemotherapeutic drugs. Furthermore, treatment of the cells with mortalin shRNA or inhibitors reverted the drug resistance of cells and dampened their migration and invasion potentials.

## Results and Discussion

### Mortalin-overexpressing cells possess higher level of expression of cancer cell stemness markers

**Figure 1:**
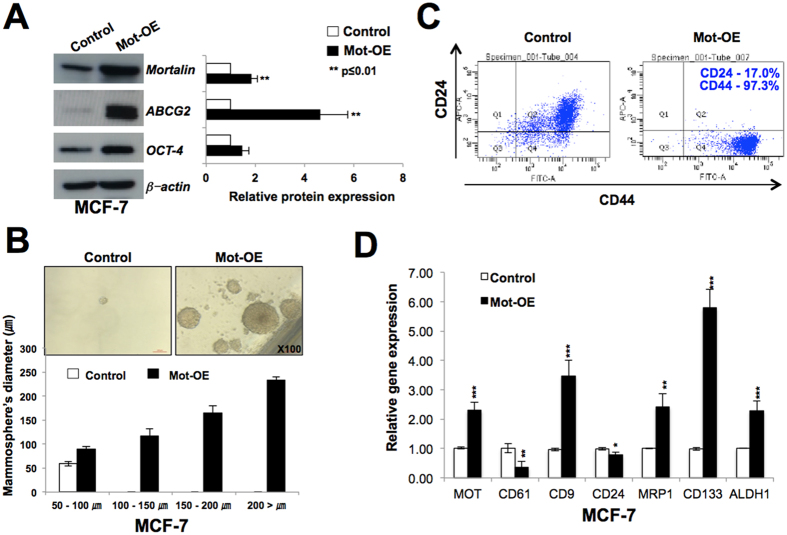
Mortalin-overexpressing MCF-7 cells show cancer stem cell characteristics. Mortalin-overexpressing MCF-7 cells showed higher expression of cancer stem cell marker proteins, ABCG2, OCT-4, as determined by Western blotting (**A**) and high degree of spheroid formation (**B**). FACS and RT-qPCR analyses revealed higher level of CD44 (**C**), CD9, MRP1, CD133 and ALDH1 (**D**) expression and low level of CD24 (**C**,**D**) and CD61 (**D**) expression in mortalin-overexpressing cells as compared to the control.

**Figure 2:**
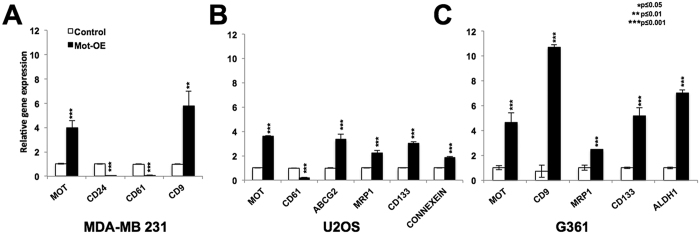
Mortalin-overexpressing MDA-MB 231, U2OS and G361 cells show cancer stem cell characteristics. Mortalin-overexpressing MDA-MB 231 cells show low level of CD24 and CD61 expression and high level of CD9 expression as compared to control cells (**A**). Mortalin-overexpressing U2OS cells showed low level of expression of CD61 and high level of expression of ABCG2, MRP1, CD133 and connexein as compared to control cells (**B**). Mortalin-overexpressing G361 cells showed high level of expression of CD9, MRP1, CD133 and ALDH1 (**C**).

Mortalin is enriched in a large variety of cancer cells^15^,^27^,^28^,^29^,^30^,^31^. In the present study, we first investigated the expression level of mortalin and CD24 in parallel in normal, immortalized and tumor derived cells (Supplementary Fig. 1A). As expected, mortalin was upregulated in all the cancer cell lines examined as compared to the normal cells. Interestingly, CD24 expression showed variability. Whereas SV40-immortalized fibroblasts (JFCF-6B and -4D) and several tumor-derived cells (MCF-7, G361, SKOV3, HUH-6, A549, DLD1, COLO 320, HCT 116) showed increase in CD24 expression as compared to the control cells, others (MDA-MB 231, Saos-2, HeLa, HUH-7, H1299) (Supplementary Fig. 1A) showed decrease. Based on these data, we selected breast adenocarcinoma, MDA-MB 231 (low level of CD24) and MCF-7 (high level of CD24), for the current study and determined the role of mortalin by generating their overexpressing derivatives. In order to examine the role of mortalin in cancer cell stemness characteristics, we first investigated the expression of two major stem cell markers, ABCG2 and OCT-4 in control and their mortalin-overexpressing derivatives (Mot-OE) by Western blotting using specific antibodies. As shown in Fig. 1A, Mot-OE MCF-7 cells possessed higher expression of both ABCG2 and OCT-4 as compared to the control and showed high efficacy of spheroid formation (Fig. 1B). Consistent with these, Mot-OE cells exhibited CD44^high^/^+^ (97.3%) and CD24^low/−^ (17.0%) level of expression as compared to the parent MCF-7 cells (Fig. 1C). The results were also validated by RT-qPCR that confirmed higher level of expression of CD9, MRP1, CD133 and ALDH1 (Fig. 1D) in Mot-OE cells as compared to the control. Furthermore, mortalin overexpression caused decrease in CD61 and CD24 expression at the transcription level (Fig. 1D). Mortalin-overexpressing MDA-MB 231 cells also showed higher level of expression of CD9 and lower level expression of CD24 and CD61 as compared to the control parental cells (Fig. 2A). These cells also showed high spheroid-forming capability (Supplementary Fig. 2A), about 10-fold higher expression of cancer stem cell marker, CK19 (Fig. 2B) and significant upregulation of ABCG2 and OCT-4 (Supplementary Fig. 2C). Similar results were obtained in U2OS (Fig. 2B and Supplementary Fig. 2D) and G361 (Fig. 2C) cells showing that mortalin upregulation enhances the cancer stem cell characteristics as determined by upregulation of ABCG2, MRP1, CD133, and downregulation of CD61 and CD24.

Examination of cell migration characteristics of control and Mot-OE cells revealed that MCF-7/Mot-OE cells possess higher migration and invasion ability (Supplementary Fig. 3A,C) and was consistent with the earlier findings^22^. Furthermore, both migration and invasion were compromised in cells treated with mortalin-shRNA as compared to the respective controls (Supplementary Fig. 3B,D). Similar results were also obtained for MDA-MB 231 cells^31^. Taken together, these data suggested that an upregulation of mortalin expression contributes to cancer cell stemness in all the cell lines examined.

### Mortalin contributes to drug resistance in cancer cells and offers a target for better cancer therapy

**Figure 3:**
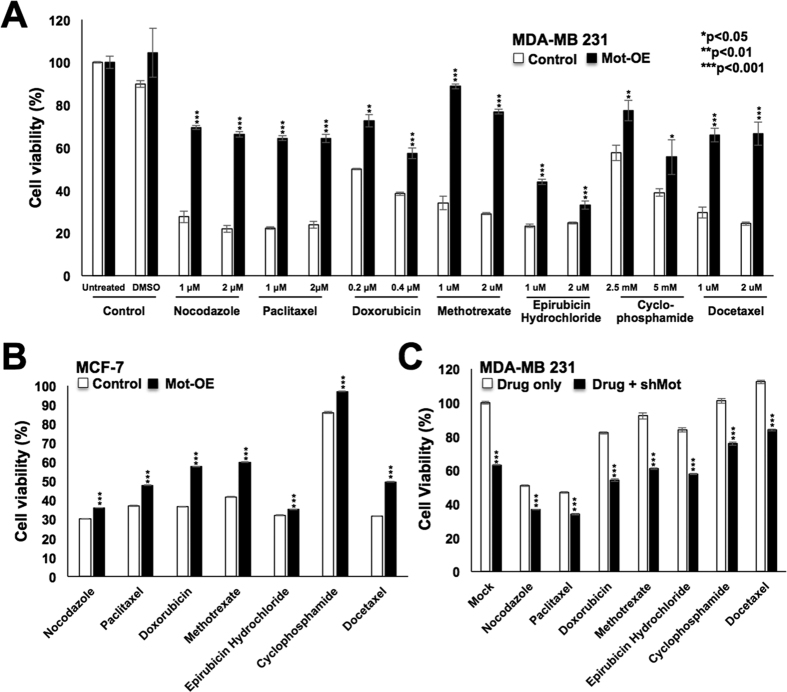
Mortalin overexpression that caused drug resistance was reverted by knockdown of mortalin with specific shRNA. Mortalin-overexpressing MDA-MB 231 (**A**) and MCF-7 (**B**) cells showed higher viability when treated with a variety of drugs indicating drug resistance characteristics. Knockdown of mortalin with shRNA sensitized the cells to drugs (**C**).

Since resistance of cancer stem cells to chemotherapeutic drugs is one of their major characteristics that possess a foremost hurdle to cancer treatment, we next investigated if mortalin overexpression contributes to drug resistance in cancer stem cells. As shown in Fig. 3A,B, mortalin-overexpressing derivatives of both MDA-MB 231 and MCF-7 cells showed resistance to several anticancer drugs. Of note, mortalin-knockdown using shRNA plasmid (Supplementary Fig. 4A) sensitized the cells to the drugs (Fig. 3C). In order to further validate these findings, we recruited mortalin-targeting shRNA-expressing adenovirus (harboring independent mortalin targeting site as described in Materials and methods section) that was shown to cause mortalin knockdown and tumor suppression *in vitro* and *in vivo*^32^. Cells treated with sub-toxic doses of mortalin-targeting shRNA-expressing adenovirus, as described earlier^31^,^32^, showed better drug response (Supplementary Fig. 4B–D). In line with this data, Yang *et al*.^26^ have also reported that an elevated level of mortalin expression in ovarian cancer cells was responsible for their resistance to cisplatin^26^.

**Figure 4:**
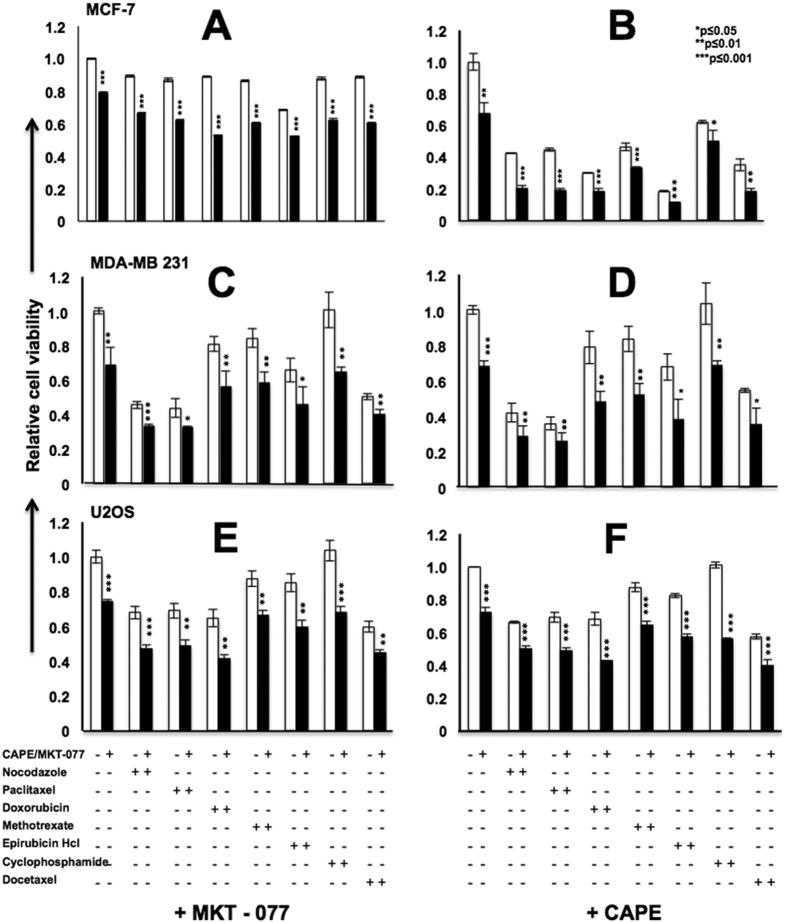
Mortalin overexpression that caused drug resistance was reverted by mortalin inhibitors. MCF-7 (**A,B**), MDA-MB231 (**C,D**) and U2OS (**E,F**) cells when treated with mortalin inhibitors (MKT-077 or CAPE) showed better response to a variety of anticancer drugs.

**Figure 5:**
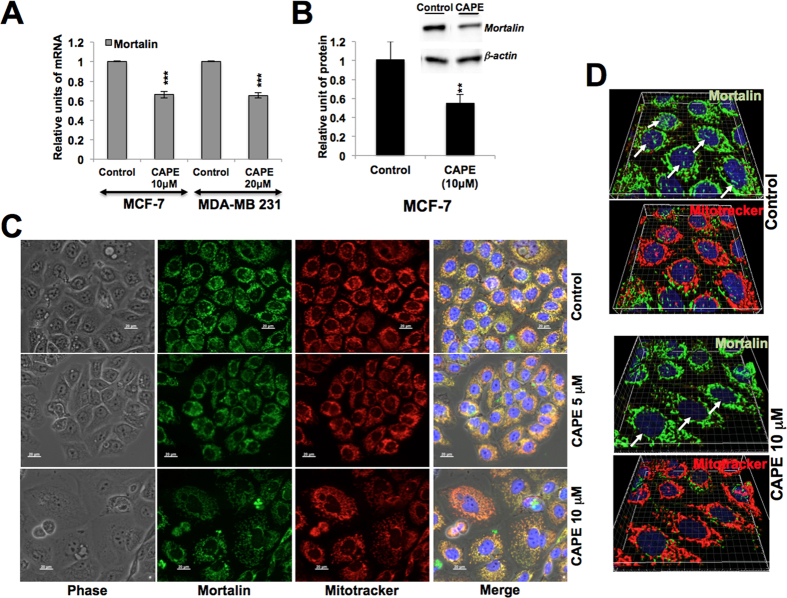
CAPE caused prominent decrease in nuclear mortalin. MCF-7 and MDA-MB 231 cells treated with CAPE showed downregulation of mortalin mRNA (**A**) and protein (**B**) as compared to untreated control cells. Co-immunostaining of control and CAPE-treated cells with mortalin antibody and mito-tracker showed decrease in mortalin intensity (**C**). High resolution confocal laser images showed remarkable reduction in nuclear mortalin, shown by white arrows (**D**).

In light of the above data, we next used mortalin inhibitors MKT-077 (a cationic rhodacyanine dye)^17^,^33^,^34^,^35^ and CAPE (Caffeic Acid Phenethyl Ester, a bioactive compound in honey bee propolis)^36^ to sensitize cancer cells to various drugs. Since MKT-077, CAPE and other drugs, used in the present study, could lead the cancer cells to apoptosis, their sub-toxic doses (as determined by independent experiments) were used in order to avoid their cytotoxic effect per se, and to achieve knockdown of mortalin as reported earlier^17^,^33^,^34^,^35^. As shown in Fig. 4A, we found that pretreatment of MCF-7 cells with sub-toxic dose of MKT-077 (0.2~0.5 μM), sensitized them to various chemotherapeutic drugs) (Table 1). CAPE (0.8 μM) that was seen to cause reduction in mortalin expression, both at the transcriptional and translational level (Fig. 5A,B) in cancer cells also sensitized them to a variety of drugs (Fig. 4B). Similar results were obtained in MDA-MB 231 and U2OS (Fig. 4C–F) cells suggesting that the targeting of mortalin by shRNA or small molecules including natural drugs, potentiated the effect of anticancer drugs.

Mortalin has been shown to play a key role in mitochondria biogenesis^21^. It has been identified as the only ATPase component of the mitochondrial import complex, essential for the translocation of most mitochondrial inner membrane and matrix proteins. It binds to Tim44 (inner mitochondrial membrane translocase) and serves as the ATP-driven force generating motor during protein import^37^,^38^,^39^. Mortalin depletion altered mitochondrial bioenergetics, depolarized mitochondrial membrane, decreases oxygen consumption and extracellular acidification and increased oxidative stress in medullary thyroid carcinoma cells^40^. We have earlier detected an expression of retrovirally-expressed mortalin in mitochondria^22^, and hence the mitochondrial functions of upregulated mortalin may contribute to increasing metabolic demand that accompanies increased proliferation capacity of cancer stem cells. Glucose uptake, ATP content and membrane potential are higher in CSC as compared to non-CSC. Analysis of metabolic curves of MCF-7 and their mortalin-overexpressing derivatives by real time automated kinetic cell assays (OmniLog Incubator) confirmed higher metabolic rate in mortalin-overexpressing derivatives (Supplementary Fig. 1B). Mitochondria has been implicated in cell stemness and differentiation properties^41^,^42^,^43^, also due to its key role in cellular oxygen consumption and extracellular acidification characteristics. Mortalin, Hsp60 and several other mitochondrial proteins have been shown to be essential regulators of oxidative stress^21^,^22^,^44^,^45^,^46^,^47^. Role of mortalin in protection against oxidative stress was also confirmed in Alzheimer disease model where suppression in mortalin expression significantly increased mitochondrial membrane depolarization and reduction in cellular ATP levels in neuroblastoma cells^48^. It was shown to regulate hematopoietic stem cell function by controlling oxidative stress^44^,^49^. In light of these reports, increased mortalin expression in cancer cells was predicted to increase (i) in mitochondrial membrane potential resulting in lower ROS level, (ii) oxygen consumption in hypoxia condition, cellular ATP levels and (iii) maintain cancer stemness and drug resistance properties.

A variety of stressful environmental conditions induce heat shock proteins. Cancer, a physiologically stressed conditions that challenge cell survival under limited nutirents and oxygen supply, has been associated with upregulation of heat shock proteins (Hsp) including mortalin, Hsp70, Hsp90 and Hsp27. These have been predicted to hold diagnostic and prognostic clinical applications^50^,^51^,^52^ and hence warranted studies. Multifunctional stress chaperone mortalin, enriched in cancers and found in various subcellular sites (the mitochondrion, plasmamembrane, endoplasmic reticulum, cytosol and nucleus), has been shown to interact with tumor suppressor protein-p53. Mortalin inactivates the transcriptional activation and apoptotic functions of p53 in cancer cells, resulting in activation of proproliferation signaling^17^,^18^,^19^,^41^,^53^,^54^,^55^. Nuclear mortalin has been shown to promote cancer cell metastasis by mechanism involving functional activation of telomerase and heterogeneous ribonucleoprotein K (hnRNP-K)^22^. Role of mortalin in cancer cell stemness has not been elucidated and hence warrated investigations. We found that core markers of cancer cell stemness (i) ABCG2, the ATP-binding cassette transporter protein that reduces the intracellular concentration of drugs and (ii) OCT-4 transcriptional factor, critically involved in the self-renewal and drug resistance^4^,^5^,^6^,^7^,^8^,^9^ were upregulated in mortalin-overexpressing cells. Furthermore, the expression level of stem cell surface marker proteins, CD133, MRP1, ALDH1, CD9, also increased in mortalin-overexpressing cells. In light of these information and the present data, we next considered if anti-mortalin small molecules, MKT-077 and CAPE, affected a specific pool of subcellular mortalin. MKT-077 has been reported to cause significant decrease in mortalin levels leading to short/compressed/fragmented mitochondria^25^,^33^,^40^,^56^,^57^,^58^. It was also shown to abrogate mortalin-p53 interactions, causing nuclear translocation and reactivation of p53 in cancer cells^17^,^18^,^33^,^35^. Pilzer *et al*.^25^ have also shown that mortalin-MAC (Membrane Attack Complex) complexes are released in membrane vesicles from complement attacked cells causing resistance of cancer cells to complement-dependent cytotoxicity^25^. siRNA or MKT-077 mediated knockdown of mortalin reduced MAC elimination, and resulted in enhanced cell sensitivity to MAC-induced cell death.

**Figure 6:**
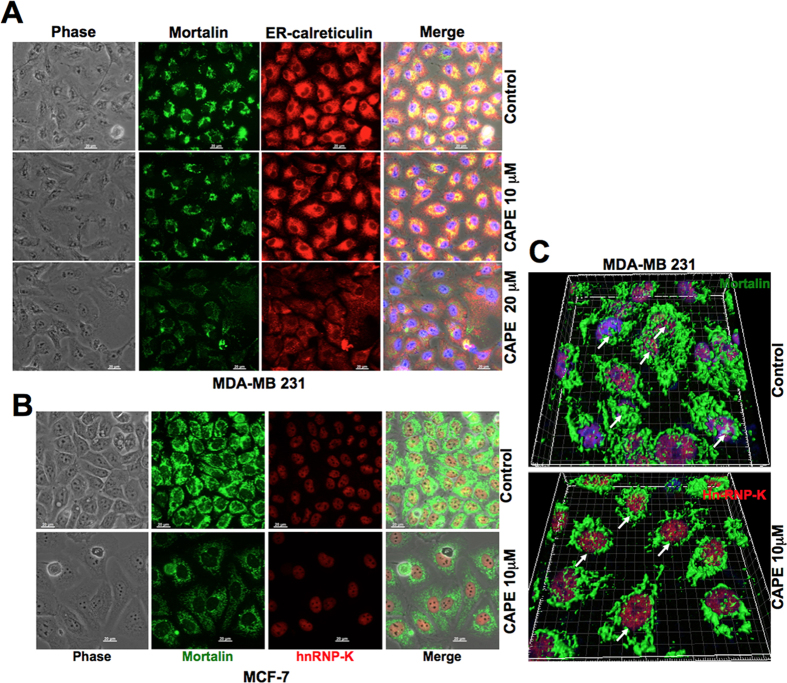
CAPE-treated cells showed remarkable decrease in nuclear mortalin. CAPE-treated MDA-MB 231 cells showed downregulation of mortalin and calreticulin. Dose response analyses showed stronger effect on mortalin than calreticulin (**A**). Co-immunostaining of mortain and nuclear protein hnRNP-K showed reduction in mortalin and its remarkable clearance from the nucleus (**B**) IMARIS images of control and CAPE-treated cells showing reduction in nuclear mortalin, shown by white arrows (**D**).

We examined the effect of CAPE on mortalin expression by Western blotting and co-immunostaining assays. As shown in Fig. 5A–D, CAPE-treated cells showed decrease in the level of mortalin expression at mRNA as well as protein level, decrease in intensity and shift in mortalin staining pattern from perinuclear to pancytoplasmic type. Furthermore, decrease in nuclear mortalin was clearly observed in high resolution images taken by confocal laser microscope (Fig. 5D). Co-immunostaining of mortalin and ER resident protein, calreticulin, in control and CAPE-treated cells revealed that although high doses of CAPE caused decrease in both the proteins, low dose was more effective to mortalin (Fig. 6A). Similar analysis with hnRNP-K revealed significant decrease in mortalin in the nucleus of CAPE-treated cells (Fig. 6B,C). Taken together, it was concluded that targeting mortalin by sub-toxic doses of CAPE caused drug sensitization by multiple ways including its functions in mitochondria, ER and nucleus^21^; nuclear clearing seemed most remarkable. Of note, nuclear mortalin has been assigned roles in EMT and malignant transformation^22^,^31^. Other drugs that target mortalin or its functions include, phytochemicals Withanone, Withaferin-A and Embelin. They cause activation of tumor suppressor protein, p53, and deactivation of metastatic signaling in cancer cells^59^,^60^,^61^, and veratridine that sensitized cancer cells to chemotherapeutic drugs by UBXN2A-dependent inhibition of mortalin^62^.

The most effective chemotherapeutic agents in breast cancer are doxorubicin, taxol, cyclophosphamide, methotrexate, 5-fluorouracil and their combination. Although each of these drugs possesses appreciable tumor regression and anti-metastatic properties, they are often complicated drug-resistant cancer cell sub-population (CSCs) responsible for tumor relapse. Vargas-Roig et al. reported increased level of nuclear Hsp70 in the clinical samples of breast cancer that showed drug resistance to doxorubicin, cyclophosphamide, methotrexate and epirubicin^49^. We have earlier demonstrated that mortalin promotes malignant transformation of cancer cells by increasing their proliferative and migration capacity and hence it is a promising target for cancer therapy^15^,^17^,^18^,^19^,^22^,^31^,^32^,^53^. In the present study, we report that the CSC have high expression of mortalin and contributes to their stemness and drug resistance characteristics. We demonstrate that anti-mortalin drugs, CAPE and MKT-077, reverted the drug resistance and hence could be useful new adjuvants for increasing the efficacy and outcome of chemotherapy.

## Materials and Methods

### Cell culture and reagents

Human normal cells (TIG-3 and MRC5) and cancer cell lines, breast cancer (MDA-MB 231, MCF-7), osteosarcoma (U2OS, Saos-2), cervical carcinoma (HeLa), hepatocellular carcinoma (HUH-6, HUH-7), ovarian carcinoma (SKOV3), adenocarcinoma (A549) and colorectal adenocarcinoma (DLD-1, COLO 320 and HCT116), procured from JCRB or DS Pharma, Japan, were cultured in DMEM (Life Technologies). Human melanoma (G361) (JCRB, Japan) was cultured in McCoy’s 5A (Life Technologies). Mortalin-overexpressing cells were generated by retroviral vector as described previously^15^,^22^. Mortalin-targeting adenovirus expressing mortalin shRNA (#009-GAATGA GGCTAGACCTTTA) was generated and used as described earlier^32^. Plasmid based mot-shRNA 2166 (5′-ACCATCTCGCACACAGCAATTCAAGAGATTGCTGTGTGCGAGATGGTT-3′) was constructed and used as described earlier^18^. Chemotherapeutic drugs were purchased from Sigma (Nocodazole, Paclitaxel, Doxorubicin hydrochloride, Cyclophamide monohydrate) or Wako (Methotrexate, Epirubicin hydrochloride and Docetaxel). Anti-mortalin antibodies (polyclonal and monoclonal) were raised in our laboratory. Mortalin targeting shRNA-expressing adenovirus were generated as described earlier^32^.

### Cell proliferation assay

Cytotoxicity and cell survival were assessed using MTT {3-(4,5-dimethylthiazol-2-yl)-2,5-diphenyltetrazolium bromide} (Life Technologies) assays in which the cell viability was estimated by the conversion of yellow MTT by mitochondrial dehydrogenases of living cells to purple formazon (MTT assay)^22^. Statistical significance of results was determined from 3–4 independent experiments including triplet or quadruplet sets in each experiment.

### Cell migration and invasion assay

Cells migration and invasion assays were performed using Transwell chamber (Corning, NY) and BD BioCoat Matrigel Invasion Chamber (BD Bioscience, MA), respectively, as described earlier^22^.

### Western blotting

Cells were cultured in DMEM (5% fetal bovine serum) in 100-mm plates and lysed using 1% Nonidet P-40 buffer containing a protease inhibitor cocktail (Sigma Aldrich). The protein concentrations of whole cell lysate were measured by bicinchonic acid assay (BCA) (Thermo Fisher Scientific, Rockford, IL). The cell lysates (10–20 μg) were separated in 10% SDS-polyacrylamide gel electrophoresis (SDS-PAGE) and transferred to a polyvinylidene difluoride (PVDF) membrane (Millipore, Billerica, MA). Blocked membranes were probed with target protein-specific primary antibodies, ABCG2 (Novus Biologicals, Littleton, CO) and OCT-4 (Cell signaling, Beverly, MA), CD24 (Santa Cruz Biotechnology INC. CA, United States), Anti-Calreticulin (Abcam, Cambridge UK) overnight at 4 °C. The blots were incubated with the following secondary antibodies conjugated to horseradish peroxidase: anti-rabbit IgG and anti-mouse IgG (Cell signaling technology) and developed by enhanced chemiluminescence reaction (ECL) (Elpis Biotech, Daejeon, Korea).

### Immunofluorescence staining

Cells were fixed on a glass coverslip placed in a 12 well culture dish with 4% paraformaldehyde in PBS, permeabilized with 0.1% Triton X-100 for 10 mins, blocked with 0.2% BSA/PBS for 1 h and were then incubated with specific antibody (as described above) for overnight at 4° C. For mitochondrial staining, MitoTracker^®^Red CMXRos (Invitrogen, M7512) was added into the cell culture media at final concentration of 50 nM. The cells were incubated under normal culture condition for 20 mins then visualized by Carl Zeiss microscope (Axiovert 200 M, Tokyo, Japan). Counter staining was performed with Hoechst 33342 (Sigma) for 10 mins in dark. To rule out the artifacts and examine the signal in more details, z-stacks were acquired with confocal laser scanning microscopy (Zeiss LSM 700). The files were transferred to a graphic workstation and analyzed with IMARIS software (Bitplane, Zurich, Switzerland).

### Flow cytometric analysis

For the assessment of cancer stem cell maker expression, cells were collected and suspended at a density of 1 × 10^6^ cells/ml. The cells were stained with fluorescein isothiocyanate (FITC)-conjugated anti-CD24, allophycocyanin (APC)-conjugated anti-CD44, or phycoerythrin (PE)-conjugated anti-CD19 following the manufacturer’s instructions. Cells were incubated at 4 °C for 1 h followed by washings (twice) with PBS and analysis using a Flow cytometer (FACS Caliber, Becton Dickinson).

### Mammosphere formation assay

For mammosphere formation, cells were plated onto 6-well plates at a density of 1 × 10^3^ cells/ml in DMEM/F-12 (Hyclone, Logan, UT) supplemented with 2% B27 (Gibco, Carlsbad, CA), 10 ng/ml EGF, and 10 ng/ml FGF (ProSpec,East Brunswick, NJ). Growth factors were added to the mammosphere cultures every 3 days, and mammospheres (>40 μm in size) were counted on Day 7.

### RNA isolation and cDNA synthesis

Total RNA was extracted from MDA-MB231, U2OS and G361 cells and their mortalin-overexpressing variants using TRIzol Reagent (Life Technologies) following supplier-described protocol. Quality and quantity were determined by spectrophotometery (Nano Drop, 1000 spectrophotometer). For cDNA synthesis, total RNA (1 μg) was reverse-transcribed into cDNA using Quantitect Reverse Transcriptase Kit (QIAGEN) following the manufacturer’s protocol. cDNA was stored at −20° C for PCR.

### Quantitative real-time RT-PCR

Gene expression was quantified by quantitative real time PCR using Syber Select Master mix (Applied Biosystem, Life Technologies), gene specific primers (Table 2) and EcoTM Real-Time PCR System (Illumina, San Diego, CA) wherein the relative level of expression of target gene was normalized against the internal control 18 S by ∆C_T_ method. An amplification plot between fluorescence signals vs. cycle number was plotted. The difference between the mean values in the triplicated samples of targeted genes and internal control 18 S were calculated by Microsoft Excel and the relative quantitative value was expressed as 2^−∆C^_T_. All the experiments were performed, at least thrice, for statistical significance.

### Metabolic rate assay

MCF-7 cells and their mortalin-overexpressing derivatives were suspended in serum-free, phenol red-free and glucose-free RPMI-1640 medium containing antibiotics and glutamine (4 mM). Cells were dispensed into PM-M1 microplates (2,500 cells/50 μL per well) and incubated for 24–72 h followed by addition of Redox Dye Mix MA (10 μL). Plates were sealed with tape (LMT-SEAL-EX, Phenix Research Products, Hayward, CA) to prevent CO_2_ loss, and incubated at 37 °C in an OmniLog Incubator (Biolog, Hayward, CA) for 18–36 h to obtain kinetic record (X-axis- time and the Y-axis-OmniLog color density units) of formazan following manufacturer’s instructions.

### Statistical analysis

All the experiments were performed in triplicate. Data are expressed as mean ± SEM of triplicate experiments. Unpaired t-test (GraphPad Prism GraphPad Software, San Diego, CA) has been performed to determine the degree of significance between the control and experimental samples. Statistical significance was defined as p-value; p* < 0.05, **< 0.01, ***< 0.001 were defined as significant, very significant and highly significant, respectively.

## Additional Information

**How to cite this article**: Yun, C.-O. *et al*. Relevance of mortalin to cancer cell stemness and cancer therapy. *Sci. Rep.* **7**, 42016; doi: 10.1038/srep42016 (2017).

**Publisher's note:** Springer Nature remains neutral with regard to jurisdictional claims in published maps and institutional affiliations.

## Supplementary Material

Supplementary Files

## Figures and Tables

**Table 1:** Drug doses used for different cell lines.

	MCF-7 (μM)	MDA-MB231 (μM)	U2OS (μM)
MKT-077/CAPE	0.5/10	0.4/20	0.6/15
Nocodazole	2.0	1.0	1.0
Paclitaxel	2.0	1.0	1.0
Doxorubicin	0.4	0.2	0.2
Methotrexate	2.0	1.0	1.0
Epirubicin Hydrochloride	2.0	1.0	1.0
Cyclophosphamide	5000	5000	5000
Docetaxel	2.0	2.0	2.0

**Table 2:** Sequence of primers used for real time PCR analysis.

ABCG2	Forward	5′-TTCTCCATTCATCAGCCTCG-3′
	Reverse	5′-TGGTTGGTCGTCAGGAAGA-3′
CD61	Forward	5′-ATGGGACACAGCCAACAACC-3′
	Reverse	5′-GTGGCACAGGCTGATAATGA-3′
CD-133	Forward	5′-GCATTGGCATCTTCTATGGTT-3′
	Reverse	5′-CGCCTTGTCCTTGGTAGTGT-3′
MRP1	Forward	5′-TCTGGGACTGGAATGTCACG-3′
	Reverse	5′-CCAGGAATATGCCCCGACTTC-3′
Connexin 43	Forward	5′-CTACTCAACTGCTGGAGGGAAG-3′
	Reverse	5′-GGCCACCTCAAAGATAGACTTG-3′
ALDH 1	Forward	5′-CGCAAGACAGGCTTTTCAG-3′
	Reverse	5′-TGTATAATAGTCGCCCCCTCTC-3′
CD 24	Forward	5′-CCCACGCAGATTTATTCCAG-3′
	Reverse	5′-GACTTCCAGACGCCATTTG-3′
CD 9	Forward	5′-ATGATGCTGGTGGGCTTC-3′
	Reverse	5′-GCTCATCCTTGGTTTTCAGC-3′
Mortalin	Forward	5′-AGC TGG AAT GGC CTT AGT CAT-3′
	Reverse	5′-CAG GAG TTG GTA GTA CCC AAA TC-3′
CD61	Forward	5′-ATGGGACACAGCCAACAACC-3′
	Reverse	5′-GTGGCACAGGCTGATAATGA-3′
