# Supplementary material for: Relevance of mortalin to cancer cell stemness and cancer therapy
Source: Sci Rep. 2017 Feb 6;7:42016. doi: 10.1038/srep42016 (PMC5292728; doi:10.1038/srep42016)
Supplement: Supplementary Files [file srep42016-s1.pdf]

Relevance of mortalin to cancer cell stemness and cancer therapy

Chae-Ok Yun[1], Priyanshu Bhargava[2,3], Youjin Na[1], Jung-Sun Lee[1], Jihoon Ryu[2], Sunil C Kaul[2*] & Renu Wadhwa[2*]

[1]Department of Bioengineering, College of Engineering, Hanyang University, 222 Wangsinmi-ro, Seongdong-gu, Seoul, Republic of Korea. [2]DBT-AIST International Laboratory for Advanced Biomedicine (DAILAB), Biomedical Research Institute, National Institute of Advanced Industrial Science & Technology (AIST), Central 5-41, 1-1-1 Higashi, Tsukuba - 305 8565, Japan. [3]Graduate School of Life & Environmental Sciences, University of Tsukuba, Ibaraki- 305-8575, Japan

Running title: Mortalin contributes to cancer cell stemness

Correspondence and requests for materials should be addressed to:

Renu Wadhwa, National Institute of Advanced Industrial Science & Technology (AIST), Central 5-41, 1-1-1 Higashi, Tsukuba - 305 8565, Japan. Tel: 81-29-861-9464; Fax: 81-29-861-2900. E-mail: renu-wadhwa@aist.go.jp

Or

Sunil C. Kaul, National Institute of Advanced Industrial Science & Technology (AIST), Central 5-41, 1-1-1 Higashi, Tsukuba - 305 8565, Japan. Tel: 81-29-861-6713; Fax: 81-29-861-2900. E-mail: s-kaul@aist.go.jp

A

Relative units of mortalin and CD24 expression as determined by Western blotting with specific antibodies. Endogenous β-actin was used as an internal control.				
	Cell Lines Name	Description	Mortalin expression (Relative units)	CD24 expression (Relative units)
**Normal cells**				
1	TIG	Normal Skin Fibroblast	1.0	1.0
2	MRC5	Normal Lung Fibroblast	1.0	1.0
**Immortalized cell lines**				
3	JFCF-6B	SV 40 Immortalized Fibroblast	2.2	2.1
4	JFCF-4D	SV 40 Immortalized Fibroblast	1.3	1.6
**Tumorigenic cell lines**				
5	MDA-MB 231	Breast Adenocarcinoma	1.8	0.3
6	Saos-2	Osteogenic Sarcoma	4.5	0.8
7	HeLa	Epithelioid Cervical Carcinoma	4.1	0.5
8	HUH-7	Hepatocellular Carcinoma	1.4	0.6
9	H1299	Non-Small Lung Cell Carcinoma	2.2	0.6
10	MCF-7	Breast Adenocarcinoma	1.8	1.9
11	G361	Skin Malignant Melanoma	2.1	1.4
12	SKOV3	Ovarian Carcinoma	2.2	1.5
13	HUH-6	Human hepatoblastoma	2.0	1.8
14	A549	Adenocarcinoma	3.4	2.8
15	DLD-1	Colorectal Adenocarcinoma	1.4	1.2
16	COLO320	Colorectal Adenocarcinoma	4.0	2.1
17	HCT116	Colorectal Carcinoma	6.7	2.0

B

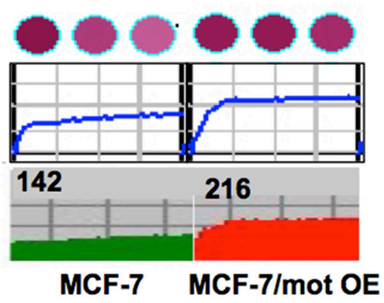

Metabolic curve (OmniLog)

Supplementary Fig. 1

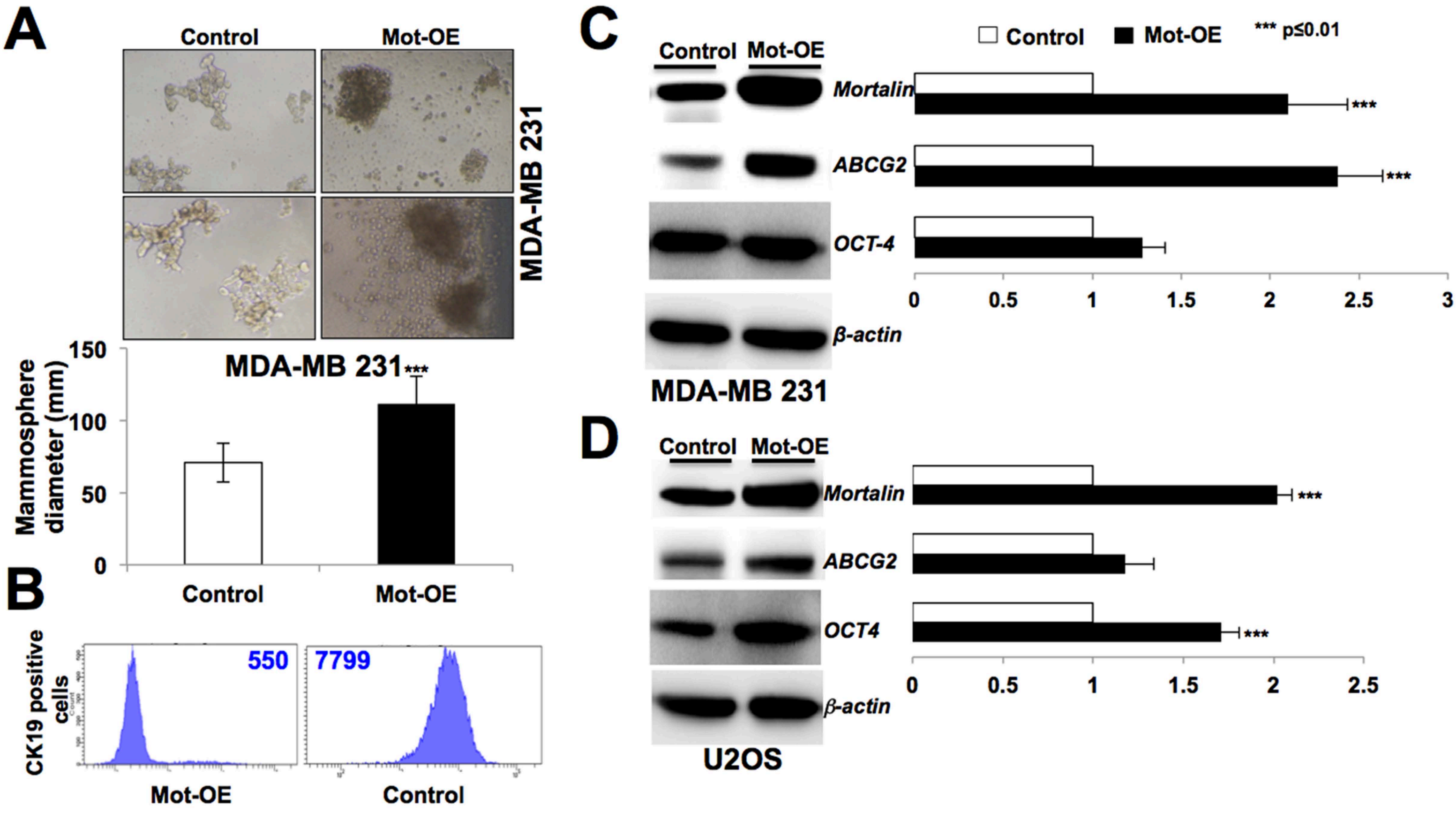

Supplementary Fig. 2

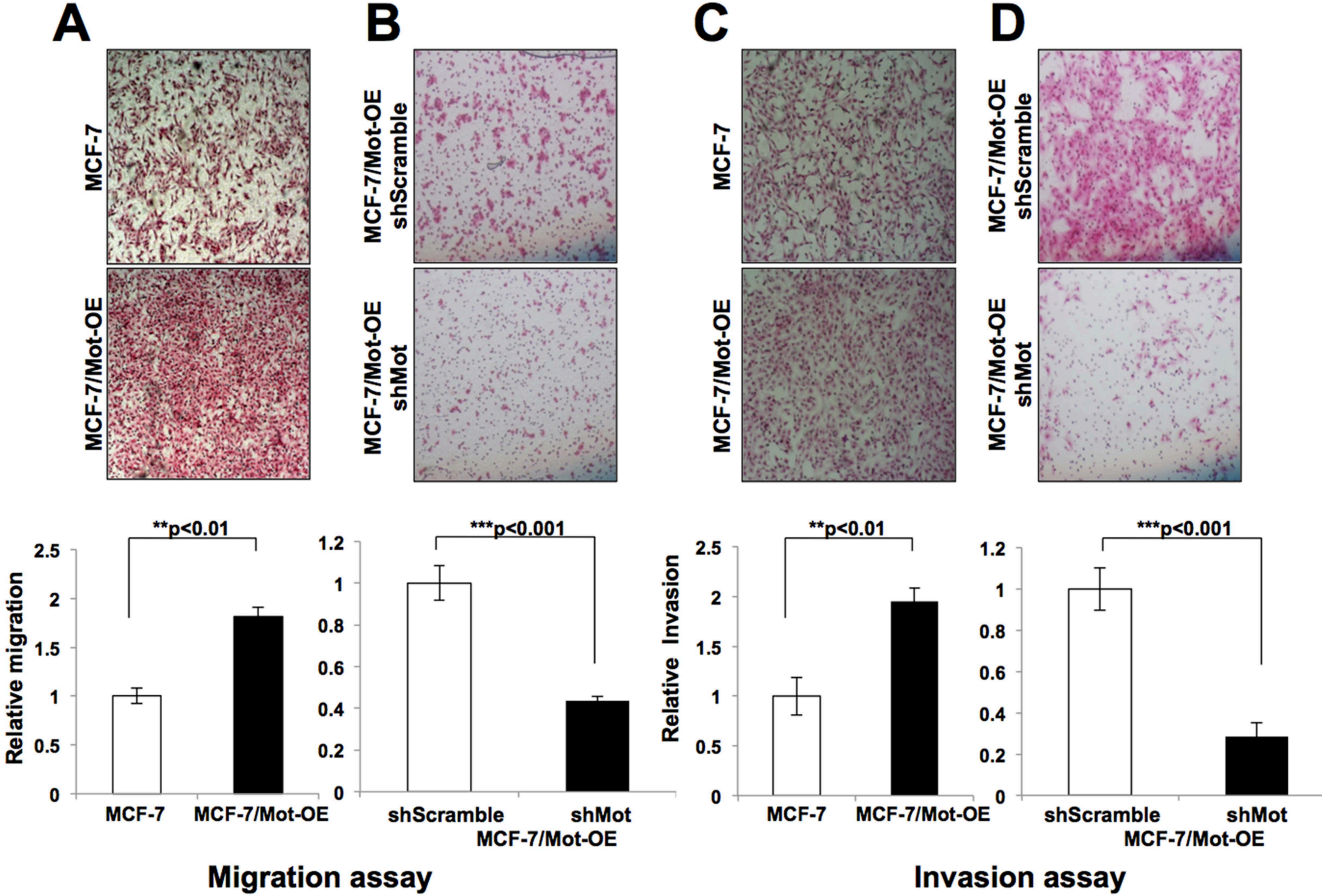

Supplementary Fig. 3

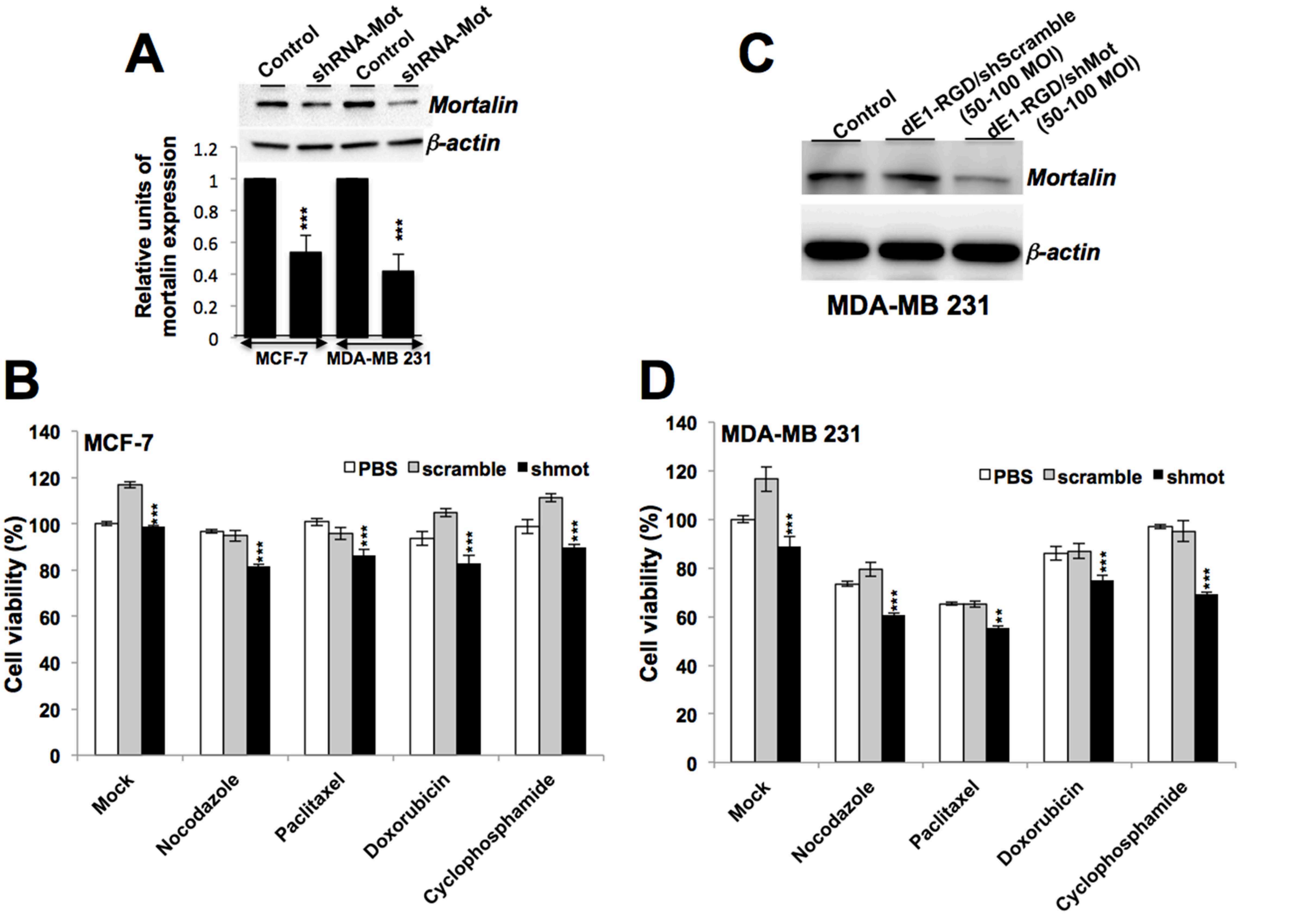

Supplementary Fig. 4

Supplementary Figure 1. Expression analyses of mortalin and CD24 in human normal and cancer cells. Quantitation of the protein signals obtained by Western blotting with specific anti-mortalin and anti-CD24 antibodies is shown. The expression was normalized against β-actin as a loading control **(A).** Real time metabolic rate of control and mortalin-overexpressing MCF-7 cells as determined by real time metabolic assay in OmniLog incubator (**B**).

Supplementary Figure 2. Mortalin-overexpressing MDA-MB 231 cells show cancer stem cell characteristics. Mortalin-overexpressing MDA-MB 231cells showed higher degree of spheroid formation expression (**A**), possessed high level of expression of CK19 (**B**), ABCG2, OCT-4 (**C**) as determined by FACS and RT-qPCR analyses, respectively. Mortalin-overexpressing U2OS cells showed high level of expression of ABCG2, OCT-4 as compared to the control cells (**D**).

Supplementary Figure 3. Mortalin knockdown in cancer cells compromised their migration and invasion capacities. Mortalin-overexpressing MCF-7 cells showed higher migration and invasion capacities as compared to control (**A** and **C**, respectively). Mortalin knockdown with specific shRNA compromised their migration and invasion capacities (**B** and **D**)

Supplementary Figure 4. Mortalin knockdown sensitized the cells to drugs. Mortalin knockdown by shRNA plasmid (**A**) and shRNA-expressing adenovirus (**C**) is shown. Mortalin-targeting shRNA-expressing adenovirus treated mortalin-compromised cells showed better response to a variety of drugs (**B** and **D**).
